# Informational content of two-dimensional panoramic radiographs and lateral cephalometric radiographs with respect to the bone volume of intraoral donor regions considering CBCT imaging

**DOI:** 10.1186/s12903-022-02344-6

**Published:** 2022-07-30

**Authors:** Phillipp Brockmeyer, Bernhard Wiechens, Tayhan Sevinc, Henning Schliephake, Wolfram Hahn

**Affiliations:** 1 Department of Oral and Maxillofacial Surgery, University Medical Center Goettingen, Robert-Koch Str. 40, D-37075 Goettingen, Germany; 2 Department of Orthodontics, University Medical Center Goettingen, Goettingen, Germany; 3 Private Office, Bonn, Germany; 4 Private Office, Goettingen, Germany

**Keywords:** Panoramic radiograph, Lateral cephalometric radiograph, Cone-beam computed tomography, Cephalometry, Intraoral bone donor sites, Augmentation, Implant

## Abstract

**Background:**

To test the hypothesis that cephalometric parameters in two-dimensional routine dental radiographs correlate with the bone volume of intraoral bone donor sites.

**Methods:**

One-hundred and eight radiographs [36 panoramic radiographs (PRs), 36 lateral cephalometric radiographs (LCRs), and 36 cone-beam computed tomography scans (CBCT)] of 36 patients (all three imaging techniques applied according to the needs of treatment planning), were analyzed individually. Cephalometric parameters (PR and LCR) were correlated with the bone volume measurement in three-dimensional CBCT scans in three intraoral donor sites (chin, mandibular retromolar region, and zygomatic alveolar crest).

**Results:**

The mean bone volumes measured for the chin were (3.10 ± 1.11 cm³ SD), the mandibular retromolar region (1.66 ± 0.54 cm³ SD), and the zygomatic alveolar crest (0.17 ± 0.04 cm³ SD). Cephalometric parameters were significantly correlated (all *p*-values < 0.05) with the bone volume in the chin and the mandibular retromolar region. The bone volume of the zygomatic alveolar crest exhibited no correlations (*p* > 0.05) with cephalometric parameters. However, it was significantly correlated (*p* < 0.01) with the mandibular retromolar bone volume. No gender-specific differences (*p* > 0.05) were observed concerning bone volumes in all bone harvesting regions. Nevertheless, the male population’s interforaminal distance in the chin region was significantly higher (*p* = 0.001).

**Conclusions:**

PRs and LCRs can be used at the initial stage of peri-implant augmentation planning to deduce conclusions about the bone volume in different intraoral bone donor sites. It can help describe indications and justify additional diagnostic options, such as three-dimensional radiologic techniques.

**Supplementary Information:**

The online version contains supplementary material available at 10.1186/s12903-022-02344-6.

## Background

Implant dentistry plays an important role in the rehabilitation of masticatory function [1]. However, sufficient bone volume and density are required for implant placement [2]. Critical bone loss occurs within the first year after tooth loss [3]. This bone reduction is particularly pronounced in the coronal third of the alveolus and on the vestibular half of the alveolar ridge [4], and it is more evident in the molar region [5]. Therefore, additional bone augmentation is often required [6].

Various bone substitute materials with varied origins are readily available [7]. Most substitute materials function as osteoconductive scaffolds for vascular and bone-forming cell incorporation [8]. As osteoconductive bone formation occurs comparatively slowly and originates mainly from the margins adjacent to the vital bone, autogenous bone grafting is still the gold standard for intraoral augmentation [8]. Vital osteoprogenitor cells and osteoblasts are transplanted with the harvested bone tissue and cause homogeneous new bone formation [8].

Harvesting bone from distant donor regions, including the iliac crest, is related to additional harvesting morbidity. Therefore, intraoral donor sites are particularly suitable for smaller bone volumes [9]. Several intraorally achievable donor sites, such as the chin region, the spina nasalis, the zygomatic alveolar crest, the lateral cortical bone of the mandible, and the mandibular retromolar region, are suitable for bone harvesting [10]. Three-dimensional radiographs [e.g., cone-beam computed tomography (CBCT) scans] are often required for implant planning and adequate assessment of bone volume in these donor sites [11]. However, additional radiation exposure of the patients accompanies these approaches [12, 13]. Two-dimensional radiographs, including panoramic radiographs (PRs) and the lateral cephalometric radiographs (LCRs), are often already available during the initial consultation prior to implant planning [12]. Per these images, bone evaluation can be performed, providing detailed references about the intraoral bone supply at varied donor regions at the beginning of treatment planning.

Therefore, this study aimed to conduct a cephalometric analysis of two-dimensional radiographs (PRs and LCRs) and correlate these data with the bone volume measured in three-dimensional radiographs (CBCT scans) in three different bone harvesting regions (chin, mandibular retromolar region, and zygomatic alveolar crest).

## Materials and methods

### Patients

This retrospective clinical trial was performed under the principles of the Declaration of Helsinki and was approved by the local Ethics Committee of the University Medical Center Goettingen (vote number: DOK_342_2015). Informed consent was obtained from all participants and/or their legal guardian(s).

A sample size of 34 participants was determined using G * Power software (v. 3.1.9.2, University of Düsseldorf) using a significance level of 0.05, a power of 0.9, and a large effect size of 0.5. The effect size was calculated for clinically relevant correlations between imaging modalities of at least r = 0.5 [14]. To ensure significance, a total of 36 patients were included in this trial, contrary to the sample size calculation (*n* = 34). No prior pilot study was conducted.

Patients comprised 20 women and 16 men, ranging in age from 18 to 51 years (mean age of 25.8 years). Radiological analysis of 108 imaging exams (36 PRs, 36 LCRs, and 36 CBCT scans) was performed. All 36 patients presented for dysgnathia consultation; therefore, results of all three imaging modalities (PR, LCR and CBCT) were available to analyse dental, dento-alveolar and skeletal configurations based on the existing orthodontic diagnosis with justified clinical indication. The scatter range of all available cephalometric parameters was within the normal spectrum and their deviations of a normal population. Imaging was not directly related to the present trial. There were no missing data. There were no special criteria for imaging selection, and no radiographs were excluded from the analysis.

PRs (14.1 s, 62–66 KVP, and 14–16 mA) and LCRs (9 s, 77–80 KVP, and 14–15 mA) were obtained using an Orthophos XG Plus X-ray Unit (Sirona Dental Systems GmbH, Bensheim, Germany). The unit was calibrated according to the manufacturer’s specifications. A 1.25× magnification was preset by the manufacturer and was applied to each patient. Patients were placed 1.5 m from the unit. To avoid PR distortions and to guarantee reliable RT1 and RT7 values, the head of each patient was positioned in the Orthophos XG Plus unit by the same radiology assistant according to the manufacturer’s instructions. In short, the chin was placed in the positioning tray, and the patient was instructed to bite with the anterior teeth into the bite splint provided for this purpose. The unit was then aligned using the Frankfurt horizontal plane.

CBCT scans were obtained using a PaX Zenith 3D (Orange Dental, Biberach an der Riß, Germany; field of view (FoV) of 240 × 190 mm, 24 s, 0.3 voxels, 120 KVP, and six mAs).

All imaging data were collected from March 2019 to July 2020. Study inclusion criteria included completed skeletal maturity and a circumferent supported natural dentition. The exclusion criteria were craniofacial syndromes, already performed maxillomandibular advancement, bone trauma, and diseases of the jaw bases.

### Image evaluation

Two blinded independent investigators (dentists) with certified expertise to perform and evaluate the applied imaging techniques evaluated the data. Investigators’ data were tested for normality using Shapiro–Wilk tests, and interrater reliability was confirmed using Bland–Altman plots (Table 1).

**Table 1:** Results of Bland–Altman plots on PR, LCR, and CBCT evaluation of both independent investigators

Parameter	Lower limit of agreement	Upper limit of agreement
Ang. Mand. [°]	− 1.85	1.89
SNA [°]	− 1.14	1.04
SNB [°]	− 1.51	2.41
ANB [°]	− 3.25	2.49
ML-NSL [°]	− 1.55	1.78
NL-NSL [°]	− 0.92	1.19
ML-NL [°]	− 1.44	1.4
NSBa [°]	− 3.91	3.44
Index [%]	− 2.31	1.84
Gn-Go-Ar [°]	− 2.06	2.04
Hasund [score]	− 1.39	1.34

### Panoramic radiograph (PR) analysis

**Fig. 1:**
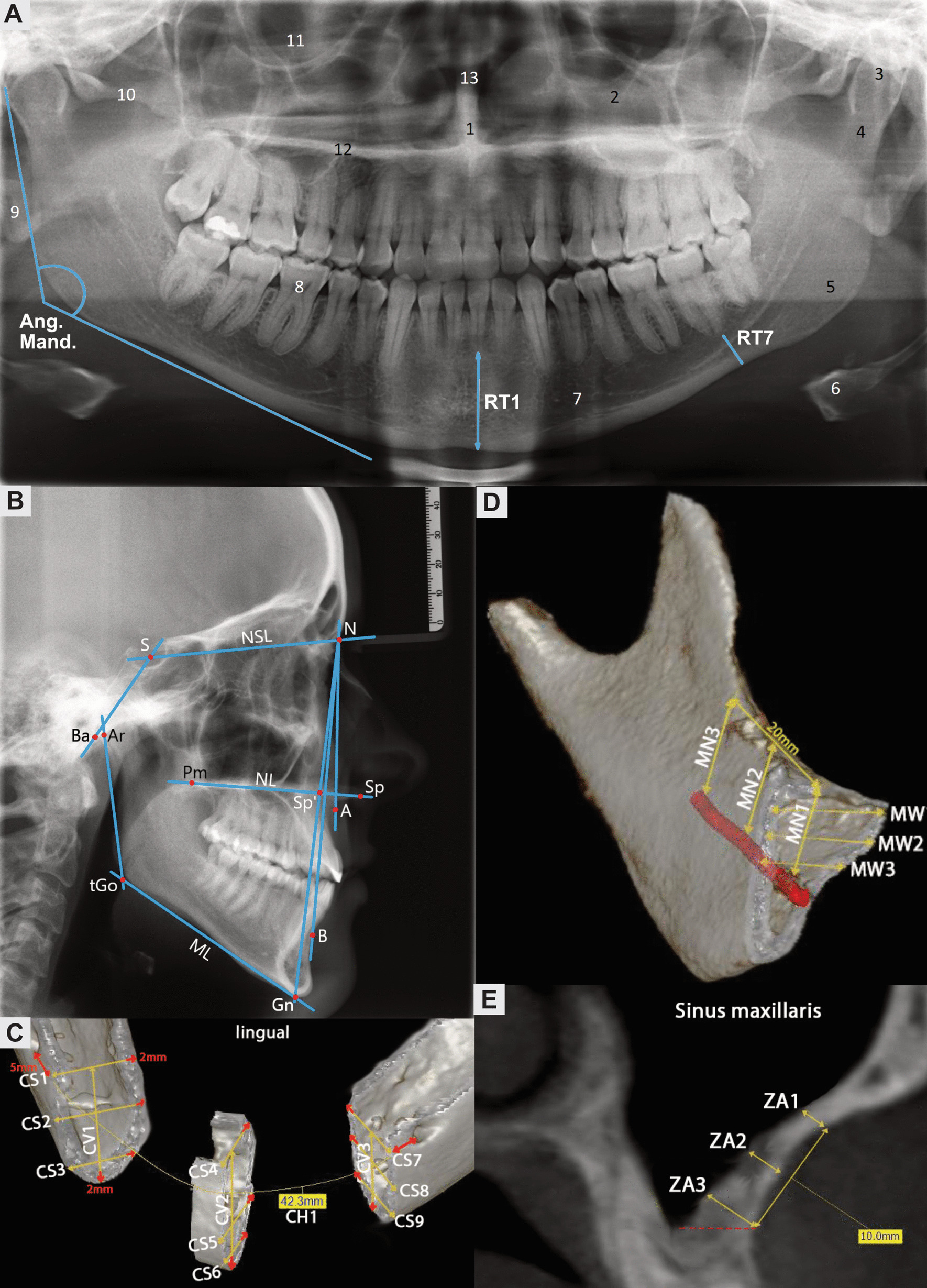
**A** PR with acquired measurements. Distance of the root tip of the central incisor (RT1) and second molar (RT7) to the mandibular base, jaw angle (Ang. Mand.), spina nasalis anterior (1), sinus maxillaris (2), caput mandibulae (3), collum mandibulae (4), mandible (5), os hyoideum (6), foramen mentale (7), bony auditory meatus (9), arcus zygomaticus (10), orbita (11), palatum durum (12), septum nasi (13). **B** LCR with cephalometric parameters. SNA angle, SNB angle, ANB angle, ML-NSL angle, NL-NSL angle, ML-NL angle, NSBa angle, Gn-Go-Ar angle, index = $\frac{{N - Sp'}}{{Sp' - Gn}} \times 100$. **C** Three-dimensional CBCT reconstruction of the chin region showing all measured distances (yellow) and safety margins (red). **D** Three-dimensional CBCT reconstruction of the right mandibular retromolar region with inferior alveolar nerve (red) and measured dimensions of the bone block. For visualization purposes, only the measurements of the first of three sections was presented. **E** Coronal CBCT cross-section of the zygomatic alveolar crest with measurements and floor of the maxillary sinus as caudal border of the bone graft (red dotted line)

All PRs were evaluated using SIDEXIS XG^®^ software (Sirona Dental Systems GmbH, Bensheim, Germany). In each PR, the distance of both root tips of the first mandibular incisors and the second mandibular molars to the base of the mandible was measured (Fig. 1A). The mean values for both central incisors (RT1) and root tips of the second mandibular molars (RT7) were obtained. Subsequently, the quotient of both distances was calculated (RT1/RT7), and the jaw angle was determined in each PR (Ang. Mand.).

### Lateral cephalometric radiograph (LCR) analysis

The entire cephalometric analysis was performed using Ivoris^®^ analyse software (Computer Konkret AG, Falkenstein, Germany).

In each LCR, eight different angles were measured, as shown in Table 2; Fig. 1B. The ratio of the anterior facial heights (Index) was determined using Hasund’s cephalometric analysis. Structural analysis of the mandible was performed according to the method proposed by Björk and modified by Segner and Hasund (Table 2) described before [15, 16].

**Table 2:** Descriptive statistics of PR, LCR and CBCT evaluation

Parameter	*N*	Mean value	Minimum	Maximum	SD
RT1 [mm]	36	25.39	14.37	34.02	4.80
RT7 [mm]	36	13.19	7.50	20.53	3.28
RT1/RT7 [mm]	36	2.02	0.90	2.95	0.52
Jaw angle [°]	36	127.50	111.08	140.40	7.20
SNA [°]	36	80.16	68.70	90.90	5.26
SNB [°]	36	78.16	62.70	94.25	8.28
ANB [°]	36	1.88	− 14	12.60	6.23
ML-NSL [°]	36	33.26	10.05	51.25	10.43
NL-NSL [°]	36	9.11	1.20	21.55	4.55
ML-NL [°]	36	24.15	2.15	42.65	9.22
NSBa [°]	36	131.89	117.85	168	9.30
Gn-Go-Ar [°]	36	125.33	103.05	138.95	8.57
Index [%]	36	77.36	64	101	7.97
Hasund [score]	36	2.47	− 12	17	6.96
Vchin [cm^3^]	36	3.10	1.59	6.16	1.11
Vretro [cm^3^]	36	1.66	0.69	2.83	0.54
Vcrista [cm^3^]	36	0.17	0.10	0.25	0.04

Distances are given in [mm], angles in degrees [°], index in percent [%], Hasund in [score] and bone volume of each donor region in [cm^3^]

### Cone-beam computed tomography (CBCT) analysis

All CBCT scans were analysed using Ez3D Plus^®^ software (Vatech Company, Hwaseong, Korea) on multiplanar reconstructions (MPR). Before measurements, the datasets were aligned to anatomical structures [17].

#### Chin region

Measurements of the bony chin were made by determining the horizontal and vertical bone dimensions, as shown in Fig. 1C. Specifically, a distance of 5 mm to the dental root tips and the mental foramen was maintained, and a distance of 2 mm to the lingual and caudal surfaces of the mandibular bone was maintained. Then, the distance between the root tips of the canines and the central incisors to the caudal surface of the mandible was measured (CV1–CV3). The width of the alveolar ridge was measured at three points each (CS1–CS9) perpendicular to these three vertical distances. The horizontal extent of the potential bone graft was measured at three locations (CH1–CH3), analogous to the width measurements.

#### Mandibular retromolar region

As no clear distal boundary is present in the mandibular retromolar region, the mesiodistal length was set at 20 mm for the analysis. Safe distances of 2 mm to the inferior alveolar nerve, to the second molar, and to the lingual surface of the mandibular bone were maintained. The vertical distance from the inferior alveolar nerve to the occlusal bone surface was determined at three different points (MN1–MN3). The alveolar ridge width was determined at right angles at three points each (MW1–MW9), along with the three vertical measurements. A schematic representation is presented in Fig. 1D.

#### Zygomatic alveolar crest

The symmetrically tapered envelope point was marked in the axial view of the CBCT scan to analyse the zygomatic alveolar crest (Fig. 1E). From this point, a dorsal and ventral section of 5 mm each was drawn, yielding a total graft width of 10 mm. The caudal limit of the graft was the floor of the maxillary sinus or the root tip of the molar. A craniocaudal length of 10 mm was marked starting from this plane. Along the marked area with a length and width of 10 mm, the bone thickness was measured at nine points (ZA1–ZA9). The coronal section plane was moved parallel to the turnover point of the zygomatic alveolar crest to achieve this measurement. The first three measurements were taken along the ventral border of the bone block at the cranial (ZA1) and caudal (ZA3) ends and centrally (ZA2). This procedure was repeated along the distal border (ZA7–ZA9). Three measurements were also taken along the envelope point (ZA4–ZA6).

#### Bone volume determination

Finally, the bone volumes for all three bone harvesting regions were calculated (Vchin, Vretro, and Vcrista). In the chin region (Vchin), the mean values for the vertical bone height (CV), horizontal bone width (CH), and sagittal bone depth (CS) were determined; these values were then multiplied with each other. The mean values for the distance of the inferior alveolar nerve to the occlusal bone surface (MN) and the width of the bone block (MW) were calculated and multiplied by the specified length of 20 mm to determine the volume of the retromolar bone block (Vretro). The height and width of the bone block were 10 mm each and multiplied by the average thickness of the graft (ZA). The volume determination in the zygomatic alveolar crest (Vcrista) followed the same principle.

### Statistical analysis

All data were tested for normality using the Shapiro–Wilk test. The statistical procedures were Student’s t test, chi-square test, linear regression, logistic regression with backwards removal algorithm, and Pearson correlation. Box-whisker plots and scatterplots were used for graphical representation. The significance level was α = 5%, and the software STATISTICA^®^ (StatSoft Europe GmbH, Hamburg, Germany) and SPSS^®^ (IBM Corporation, New York, USA) were used.

## Results

### Panoramic radiograph evaluation

In the PR evaluation, higher distances between the root tips of the mandibular middle incisors (RT1) and the lower mandibular base compared with the root tips of the second mandibular molars (RT7) were recorded. Overall, the average vertical bone height in the chin region was higher than that in the mandibular second molar region. However, higher standard deviations were reported for the chin region than for the molar region (Table 2).

### Cephalometric analysis

The detailed data of the cephalometric analysis in the LCRs, the indices, and the Hasund scores are presented in Table 2.

### Bone volume analysis

#### Chin region

The analysis revealed that the horizontal extent of the chin region was the highest in the middle position (CH2). The highest standard deviation for the horizontal expansion was observed at CH3. With a distance of 2 mm from the mandibular base, this location was the most caudal of the three horizontal measurement points. It was near the bony base of the chin region, seeming to be subject to higher interindividual standard deviations. The vertical bone analysis determined the highest mean values for the middle position (CV2). If a safe distance (5 mm) from the tooth apex was maintained, the chin provided more bone in the vertical dimension in the area of the lower incisors than below the canines. The analysis for the CV1 and CV3 measurement points barely showed any differences due to the lateral symmetry. The sagittal evaluation resulted in similar findings. The bone thickness below the canines was equally close in the lateral comparison owing to symmetry. On average, the highest bone thickness was measured at point CI5. The calculations for the bone volume in the chin region resulted in a mean value of 3.10 cm³ ± 1.11 cm³ SD.

#### Mandibular retromolar region

The average distance between the occlusal bone surface and the inferior alveolar nerve was the highest at the most distal measurement point (MN3). On average, the vestibular bone wall was thicker at the distal root (MR2) than at the mesial root of the second mandibular molar (MR1). The buccolingual bone thickness was higher at the mesial points (MW1–MW3) than at the distal points (MW4–MW6 and MW7–MW9). Differences were also noted in the cranio-caudal direction. Thus, the average buccolingual bone thickness halfway between the bone surface and the inferior alveolar nerve (MW2, MW5, and MW8) was higher than that in in the cranial (MW1, MW4, and MW7) and caudal measurement positions (MW3, MW6, and MW9). On average, a bone volume of 1.66 cm³ ± 0.54 cm³ SD was calculated for the mandibular retromolar region.

#### Zygomatic alveolar crest

On average, the measurement positions at the inflexion point of the zygomatic alveolar crest (ZA4–ZA6) had a greater bone thickness than those in the lateral position (ZA1–ZA3 and ZA7–ZA9). With an average of 0.17 cm³ ± 0.04 cm³ SD, the zygomatic alveolar crest had the lowest bone volume among all bone harvesting regions examined.

#### Affect of sex on measurements

The analysis revealed that sex significantly impacted the horizontal bone thickness in the chin region (at measuring points CH2 and CH3; *p* = 0.036 and *p* = 0.003, respectively). On average, female patients showed 3.44 and 6.15 mm less bone thickness than the male patients at the CH2 and CH3 measurements. Overall, no significant differences (*p* > 0.05) were noted between sexes concerning the bone volumes in all three analysed bone harvesting regions (Additional file 1: Fig. S1).

### Correlation analysis

#### Chin region

**Fig. 2:**
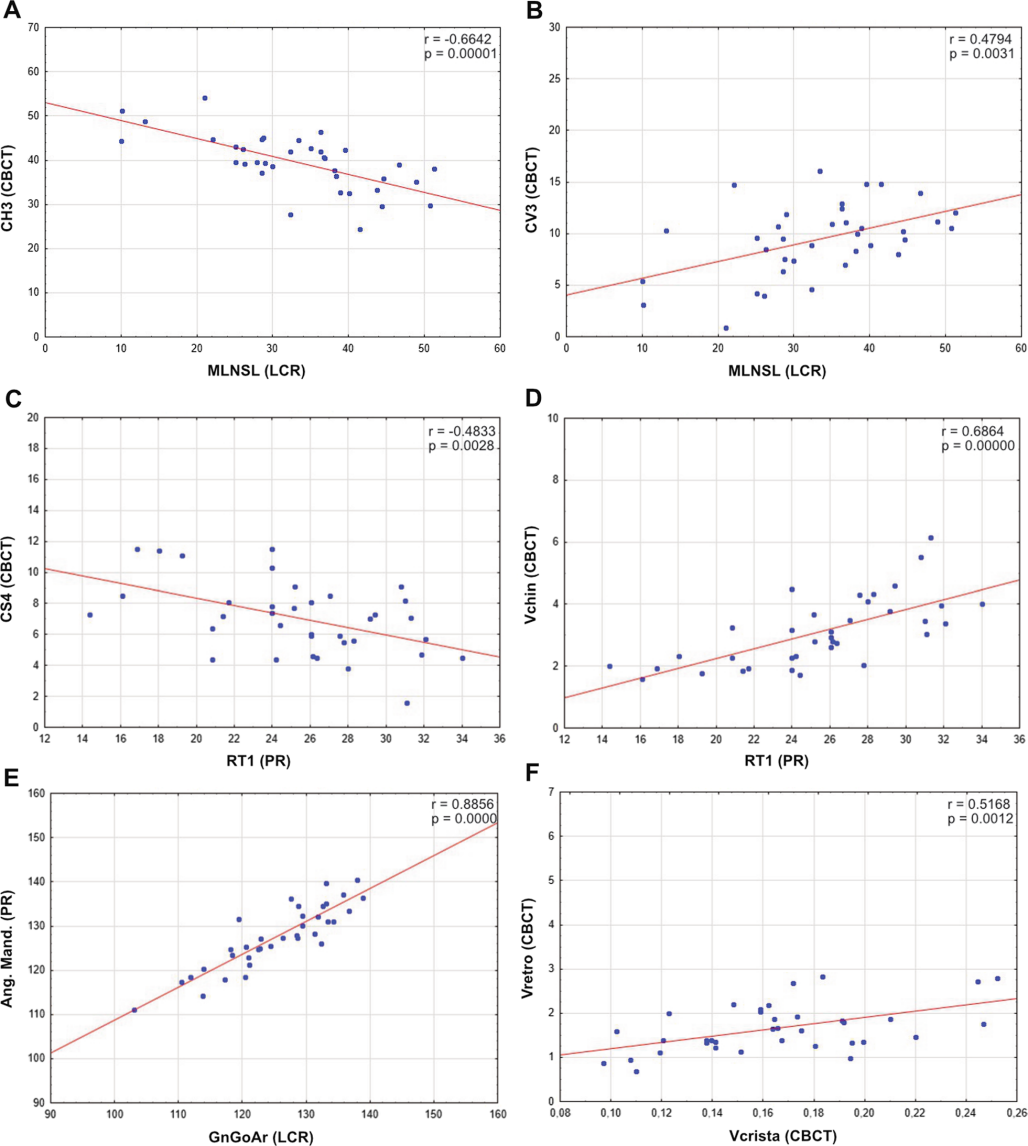
Scatterplot of Pearson correlation analysis. **A** Correlation between the ML-NSL angle (LCR; °) and the transverse width of the bony chin (CH3; CBCT; mm). **B** Correlation between the ML-NSL angle (LCR; °) and the bone height in the chin region (CV3; CBCT; mm). **C** Correlation between RT1 (PR, mm) and CS4 (CBCT, mm). **D** Correlation between RT1 (PR, mm) and bone volume of the chin region (CBCT, Vchin, cm^3^). **E** Correlation between Gn-Go-Ar angle (LCR, °) and jaw angle (PR, Ang. Mand., °).** F** Correlation between bone volume of the zygomatic alveolar crest (CBCZ, Vcrista, cm^3^) and the mandibular retromolar bone volume (CBCT, Vretro, cm^3^)

The analysis revealed a significant (*p* < 0.05) negative correlation between the ML-NSL angle (LCR) and the CH3 distance (CBCT scan) (Table 3; Fig. 2A). Thus, a larger ML-NSL angle correlates with a transversely narrower chin and, thus, a reduced interforaminal distance. Moreover, a significant positive correlation (*p* < 0.05) was observed between the ML-NSL angle and the vertical dimension of the bony chin (CV3) (Table 3; Fig. 2B). A larger ML-NSL angle correlates with an increased distance between the root tip of the mandibular incisors or canines and the mandibular base. A strong negative correlation (*p* < 0.05) between RT1 and CV2 and a significant negative correlation (*p* < 0.05) between RT1 and CS4 were found (Table 3; Fig. 2C). The sagittal extent of the bony chin cannot be evaluated in a PR. A significant positive correlation (*p* < 0.05) was noted between the RT1 measuring point and the total bone volume of the chin region (Table 2; Fig. 2D).

**Table 3:** Pearson correlation analysis between PR/LCR parameters and CBCT measurements for the chin region

PR/LCR	CBCT	Correlation coefficient	*p*-value
RT1	CV1	0.75	0.000
	CV2	0.83	0.000
	CV3	0.70	0.000
	CS4	− 0.48	0.003
	Vchin	0.67	0.000
RT7	Vchin	0.41	0.013
ML-NL	CH1	− 0.34	0.042
	CH2	− 0.44	0.007
	CH3	− 0.59	0.000
	CV1	0.44	0.008
	CV2	0.43	0.009
	CV3	0.49	0.002
	CS4	− 0.44	0.007
ML-NSL	CH1	− 0.36	0.033
	CH2	− 0.50	0.002
	CH3	− 0.66	0.000
	CV1	0.45	0.006
	CV2	0.43	0.009
	CV3	0.48	0.003
	CS4	− 0.50	0.002
Hasund	CH3	0.53	0.001
	CV1	− 0.44	0.007
	CV2	− 0.43	0.009
	CV3	− 0.47	0.004
	CS4	0.46	0.005
ANB	CV3	0.43	0.009
Gn-Go-Ar	CH3	− 0.41	0.012
Index	CV1	− 0.46	0.005
	CV2	− 0.42	0.011
	CV3	− 0.41	0.014

#### Mandibular retromolar region

A significant negative correlation (*p* < 0.05) was observed between the jaw angle (Ang. Mand.) measured on the PR and the vertical bone supply above the inferior alveolar nerve (Table 4). Thus, an increased jaw angle correlates with a reduced distance between the occlusal bone surface and the inferior alveolar nerve. The distance between the root tips of the mandibular second molars and the mandibular base measured in the PR significantly correlates (*p* < 0.05) with the vertical bone height and the bone volume measured in the CBCT scans (Table 3).

**Table 4:** Pearson correlation analysis between PR/LCR parameters and CBCT measurements for the mandibular retromolar region

PR/LCR	CBCT	Correlation coefficient	*p*-value
RT7	MN1	0.51	0.002
	MN2	0.54	0.001
	MN3	0.41	0.013
	Vretro	0.40	0.017
Jaw angle	MN3	− 0.41	0.013
SNB	MN1	− 0.34	0.040
	MN2	− 0.45	0.006
	MN3	− 0.41	0.012
ANB	MN1	0.49	0.003
	MN2	0.57	0.001
	MN3	0.34	0.044
	Vretro	0.35	0.039
Gn-Go-Ar	MN3	− 0.47	0.004
Hasund	MN3	0.50	0.002

The Gn-Go-Ar jaw angle measured in the LCR behaved analogously to the jaw angle measured in the PR concerning its significant negative correlation (*p* < 0.05) with the vertical retromolar bone supply (Table 4).

A significant positive correlation between the Gn-Go-Ar (LCR) and the jaw angle (PR) was found (r = 0.89; *p* = 0.000; Fig. 2E). The basal sagittal distal jaw relation, characterized by an increased ANB angle in the LCR, was positively correlated (*p* < 0.05) with the vertical bone supply (MN, Table 4) and the total retromolar bone volume (Vretro) (Table 4).

Similar to the chin region, specific cephalometric growth patterns and types for the mandibular retromolar bone harvesting region can be related to CBCT measurements. On the one hand, the basal sagittal distal relation (ANB angle enlarged), retrognathia (SNB angle reduced), and anterior rotations (Hasund score enlarged, and Gn-Go-Ar angle reduced) significantly correlated (*p* < 0.05) with an increased bone supply in the retromolar region (Table 4). On the other hand, an increased jaw angle was significantly correlated (*p* < 0.05) with a reduced bone supply in the mandibular retromolar region (MN3; Table 4).

#### Zygomatic alveolar crest

The analysis revealed a significant positive correlation (*p* < 0.05) between the bone volume in the zygomatic alveolar crest and the mandibular retromolar region (Table 5; Fig. 2F). No significant correlations (*p* > 0.05) were observed between the bone volume of the zygomatic alveolar crest and cephalometric parameters. A zygomatic alveolar crest with a stronger bone wall and a larger volume correlated with an increased retromolar bone supply.

**Table 5:** Pearson correlation analysis between CBCT measurements of the zygomatic alveolar crest and the mandibular retromolar region

CBCT (zygomatic)	CBCT (retromolar)	Correlation coefficient	*p*-value
ZA8	MN1	0.51	0.002
	MN2	0.45	0.007
	MW2	0.44	0.008
	MW3	0.35	0.039
	MW5	0.55	0.001
	MW9	0.35	0.039
ZA9	MN1	0.48	0.003
	MN2	0.45	0.002
	MN3	0.39	0.018
	MW2	0.34	0.044
Vcrista	Vretro	0.52	0.001

## Discussion

Augmentation of intraoral bone deficiencies is a frequent procedure [18]. Although exogenous bone substitute materials have become increasingly available, harvesting autogenous bone crafts is still considered the clinical gold standard [19]. The selection and evaluation of an ideal donor site is a crucial prerequisite. Various authors have already described it using three-dimensional radiological techniques (CBCT and computed tomography, CT) [9, 17, 20, 21]. This study examined the possibility of using routine two-dimensional dental radiographs (PR and LCR) to acquire information about the bone volume of commonly used intraoral bone harvesting regions before peri-implant augmentation. This approach can be beneficial during initial diagnostics and provides additional information before the usual three-dimensional radiological scans are performed.

Bone augmentation procedures during dental implantation are usually used in the older population after tooth loss, most likely due to periodontal reasons but also after traumatic tooth loss in younger patients. The anatomy and physiology of the jawbone substantially vary depending on patients’ age [22]. The participants’ ages in this study ranged from 18 to 51 years. However, the present study aimed to determine correlations of cephalometric parameters between standard dental radiology techniques and bone availability in different intraoral donor regions. Therefore, extrapolation to an older patient group was not performed.

The bone volumes in the three different donor regions differed significantly (*p* < 0.05) in the present analysis. While the chin region offers the most immense amount of bone for augmentation (3.10 ± 1.11 cm³ SD), only small volumes are obtainable from the mandibular retromolar region (1.66 ± 0.54 cm³ SD) and the zygomatic alveolar crest (0.17 ± 0.04 cm³ SD).

These results are comparable to those of Zeltner and colleagues [17], who described an average bone volume of 3.5 ± 1.3 cm³ SD and 1.8 ± 1.1 cm³ SD for the chin and mandibular retromolar regions, respectively [17]. The slightly higher values in the retromolar region compared to this study could be because a proportion of studied patients had distally shortened dentition [17]. Moreover, the mesiodistal length of the bone block was not determined equally for all bone grafts [17]. Thus, patients with distally shortened dentition have a larger overall bone volume in the mandibular retromolar region. These patients might be particularly suitable for harvesting compacta shells, as local atrophy reduces bone height. In contrast, the mesiodistal extent of the removable bone block increases due to the missing molars [3, 17].

However, characteristics other than bone volume may influence a surgeon’s decision when he or she is selecting the right donor site. In this investigation, various cephalometric parameters of the PR and LCR were significantly correlated (*p* < 0.05) with the three-dimensionally measured bone dimensions in frequently used bone donor sites.

A patient’s growth type could play a substantial role in the decision-making process. Patients with a vertical growth type have an increased vertical bone supply in the chin region between the apices of the incisive and the mandibular base. At the same time, a reduced interforaminal distance is expected in such patients. Moreover, the chin tends to be thinner in a sagittal direction in such patients, leading to a reduced distance to the lingual cortex. Therefore, in patients with a vertical growth pattern, the interforaminal distance and the distance to the lingual cortical bone might be more limiting factors in bone harvesting than the distance between the mandibular base and the apices of the incisors, and more bone could be harvested in the vertical dimension. Handelman also described such a narrowing of the alveolar ridge in vertical growth-type patients with the elongation of the mandibular incisors, representing a compensatory mechanism [23, 24]. Previous studies have also affirmed the involvement of the bony chin dimensions in such compensatory mechanisms [25–27]. Although the sagittal thickness of the bony chin cannot be directly assessed in PRs, a small distance between the root tip of the middle incisive and the mandibular base correlates with an extended chin in the sagittal direction. In the case of an enlarged distance between the root tip of the middle incisive and the mandibular base on the PR, the tendency to reduce the distance to the lingual cortex must be considered when removing a bone block.

Significant correlations (*p* < 0.05) were also observed between cephalometric parameters and the bone supply for the mandibular retromolar region. According to the ANB and SNB angles, patients with a distal bite position, in addition to a smaller jaw angle (Ang. Mand.), could be particularly interesting for retromolar bone block harvesting. A distal bite position could compress the retromolar space, resulting in an increased bone supply. Whether such compression possibly reduces the mesiodistal extent of the retromolar bone block was not tested in this study and should be the subject of further investigations. Compared to this study, no correlations (*p* > 0.05) existed the between ANB angle and the total mandibular retromolar bone volume, as reported previously [28, 29]. This suggests that bone volumes of individual mandibular regions correlate with cephalometric parameters, while other areas exhibit either no or opposite effects. The distance between the root tips of the second mandibular molars and the mandibular base in the PR significantly correlates (*p* < 0.05) with the mandibular retromolar bone volume. This distance likely captures the vertical dimension in the mandibular retromolar region, which is critically involved in the supplied bone volume.

The bone volume of the zygomatic alveolar crest exhibits no correlations (*p* > 0.05) with the measurements on PRs and LCRs and only correlates significantly (*p* < 0.05) with the mandibular retromolar bone volume. A plausible explanation of this observation may suggest that at least the bone thickness of the zygomatic alveolar crest correlates less with skeletal growth patterns than with distinct trajectories of masticatory loading [30]. As part of the vertical zygomatic pillar, the zygomatic alveolar crest is where masticatory pressure is transmitted and distributed [30]. These main force lines extend over the zygomatic alveolar crest and the mandibular retromolar region, especially over the linea obliqua. Corresponding thickenings of the compacta may occur in response to these force effects [30]. A strongly pronounced horizontal growth type correlates (*p* < 0.05) with increased masticatory force delivery [31]. However, this study had no statistically significant correlation (*p* > 0.05) between the Hasund score and the bone dimensions of the zygomatic alveolar crest. Further studies should be conducted to explore a possible effect between masticatory force delivery and the bone supply of the zygomatic alveolar crest.

When examining sex-specific differences in the bone supply, no statistically significant correlations (*p* > 0.05) were seen. Correlations between bone volumes and cephalometric parameters exhibited no dependence (*p* > 0.05) on sex. However, the interforaminal distance was significantly higher (*p* < 0.05) in male patients.

## Conclusions

- The use of standard dental radiographs (PRs and LCRs) in the search for suitable intraoral bone donor regions was analysed.
- Significant correlations were found between skeletal growth patterns and bone dimensions of different intraoral bone harvesting regions.
- This approach can be beneficial in the initial search for suitable donor regions and justification of further diagnostic options, such as CBCT or CT scans.

## Supplementary Information

**Additional file 1: Fig. S1.** Box-whisker plot showing bone volumes (cm^3^) in the three different harvesting regions divided by gender (female, F; male, M). Bone volume of the chin (Vchin), the mandibular retromolar region (Vretro), and the zygomatic alveolar crest (Vcrista).

## Data Availability

The datasets generated and/or analysed during the current study are not publicly available due to privacy reasons but are available from the corresponding author on reasonable request.

## Abbreviations

PR: Panoramic radiograph
LCR: Lateral cephalometric radiograph
CBCT: Cone-beam computed tomography
