# Supplementary material for: Informational content of two-dimensional panoramic radiographs and lateral cephalometric radiographs with respect to the bone volume of intraoral donor regions considering CBCT imaging
Source: BMC Oral Health. 2022 Jul 30;22:318. doi: 10.1186/s12903-022-02344-6 (PMC9339174; doi:10.1186/s12903-022-02344-6)
Supplement: Supplementary file 1 — Additional file 1: Fig. S1. Box-whisker plot showing bone volumes (cm3) in the three different harvesting regions divided by gender (female, F; male, M). Bone volume of the chin (Vchin), the mandibular retromolar region (Vretro), and the zygomatic alveolar crest (Vcrista). [file 12903_2022_2344_MOESM1_ESM.pdf]

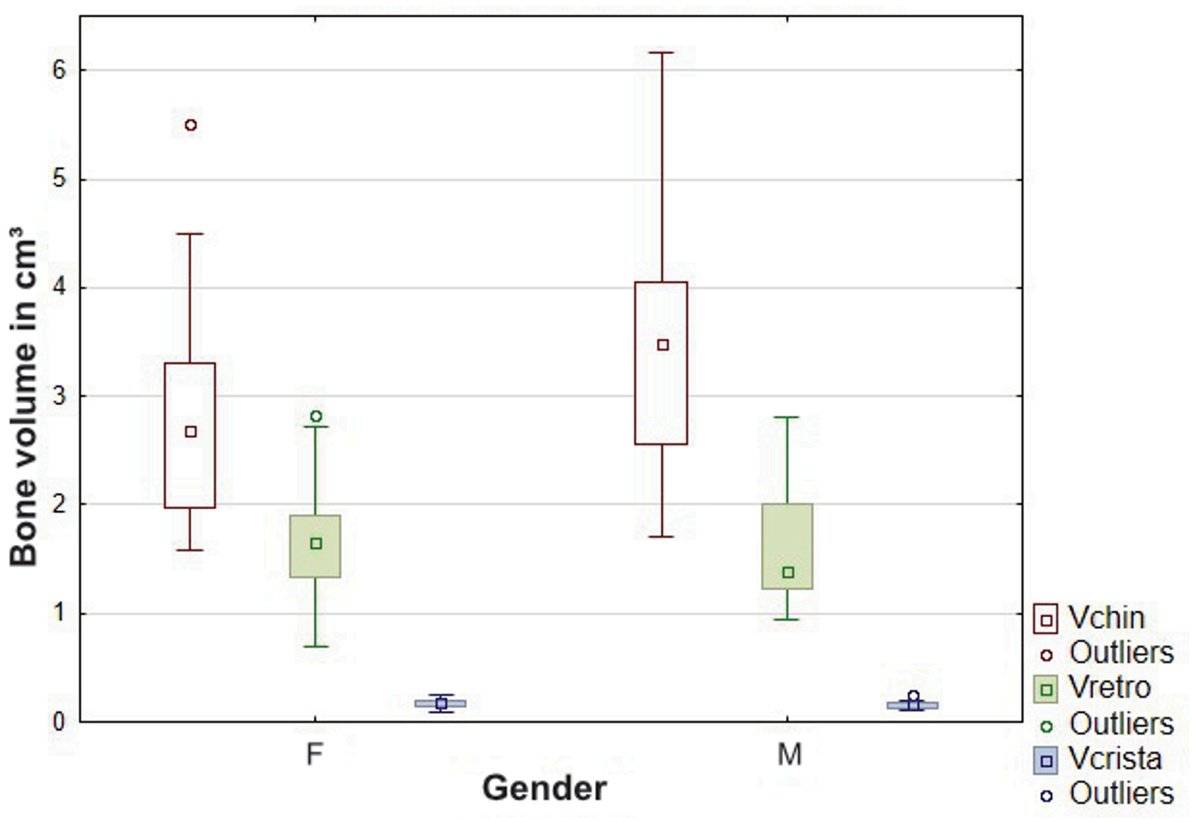

Supplementary Figure 1: Box-whisker plot showing bone volumes (cm^3) in the three different harvesting regions divided by gender (female, F; male, M). Bone volume of the chin (Vchin), the mandibular retromolar region (Vretro), and the zygomatic alveolar crest (Vcrista).
